# Long-distance electron transfer by cable bacteria in aquifer sediments

**DOI:** 10.1038/ismej.2015.250

**Published:** 2016-04-08

**Authors:** Hubert Müller, Julian Bosch, Christian Griebler, Lars Riis Damgaard, Lars Peter Nielsen, Tillmann Lueders, Rainer U Meckenstock

**Affiliations:** 1 Institute of Groundwater Ecology, Helmholtz Zentrum München, Neuherberg, Germany; 2 Section for Microbiology, Department of Bioscience, Aarhus University, Aarhus C, Denmark; 3 Department of Bioscience, Center for Geomicrobiology, Aarhus University, Aarhus C, Denmark

## Abstract

The biodegradation of organic pollutants in aquifers is often restricted to the fringes of contaminant plumes where steep countergradients of electron donors and acceptors are separated by limited dispersive mixing. However, long-distance electron transfer (LDET) by filamentous ‘cable bacteria' has recently been discovered in marine sediments to couple spatially separated redox half reactions over centimeter scales. Here we provide primary evidence that such sulfur-oxidizing cable bacteria can also be found at oxic–anoxic interfaces in aquifer sediments, where they provide a means for the direct recycling of sulfate by electron transfer over 1–2-cm distance. Sediments were taken from a hydrocarbon-contaminated aquifer, amended with iron sulfide and saturated with water, leaving the sediment surface exposed to air. Steep geochemical gradients developed in the upper 3 cm, showing a spatial separation of oxygen and sulfide by 9 mm together with a pH profile characteristic for sulfur oxidation by LDET. Bacterial filaments, which were highly abundant in the suboxic zone, were identified by sequencing of 16S rRNA genes and fluorescence *in situ* hybridization (FISH) as cable bacteria belonging to the *Desulfobulbaceae*. The detection of similar *Desulfobulbaceae* at the oxic–anoxic interface of fresh sediment cores taken at a contaminated aquifer suggests that LDET may indeed be active at the capillary fringe *in situ*.

## Introduction

Excess carbon loads in hydrocarbon-polluted aquifers rapidly lead to the depletion of electron acceptors such as molecular oxygen, nitrate and sulfate in the core of contaminant plumes, which represents a major limitation for biodegradation (Meckenstock *et al.*, 2015). This leads to steep geochemical countergradients of electron donors and dissolved electron acceptors at the plume fringes (Anneser *et al.*, 2008; Winderl *et al.*, 2008) where biodegradation is sustained by the dispersive and diffusive transport of electron acceptors from groundwater outside of the plume (Christensen *et al.*, 2000; Bauer *et al.*, 2008). The spatial separation of electron donors and acceptors represents a major limitation for microbial metabolism (Meckenstock *et al.*, 2015) because of restricted transport across such interphases. Microbial long-distance electron transfer (LDET; Nielsen and Risgaard-Petersen, 2015) could perform direct electric coupling of microbial processes across such redox gradients and would allow for a unique ecological niche in hydrocarbon-contaminated aquifers.

LDET is essentially based on the spatial segregation of redox half reactions and the presence of a conductive structure between the two locations. The concept of LDET within a geobattery was first proposed by Sato and Mooney (1960), who presented a model for the generation of subsurface electric potentials and electric fields by coupling ferrous iron oxidation and oxygen reduction by electric currents through a conductive ore body. This was extended to a biogeobattery by Revil *et al.* (2010), who suggested a microbial LDET by a conductive network of bacteria and minerals (Bigalke and Grabner, 1997; Holmes *et al.*, 2004; Naudet *et al.*, 2004; Revil *et al.*, 2010). Recently, LDET was inferred from biogeochemical profiles in marine sediments (Nielsen *et al.*, 2010; Risgaard-Petersen *et al.*, 2012). The spatial separation of the redox half reactions resulted in a characteristic pH profile (Meysman *et al.*, 2015): a pH maximum by proton consumption in the oxic zone and a pH minimum by sulfide oxidation in the sulfidic zone (Nielsen *et al.*, 2010; Risgaard-Petersen *et al.*, 2012). The LDET was shown to be mediated by long filamentous bacteria affiliated to the *Desulfobulbaceae* (Pfeffer *et al.*, 2012), bridging a suboxic zone over 1–2-cm distances where neither oxygen nor sulfide was detectable. A single filament of these so-called ‘cable bacteria' can be composed of thousands of individual cells having a characteristic, shared envelope with 15–58 marked ridges, giving them a cable-like appearance (Pfeffer *et al.*, 2012; Malkin *et al.*, 2014). Periplasmic strings underneath the ridges might serve as electric conductors with the common outer membrane as isolation (Pfeffer *et al.*, 2012; Meysman *et al.*, 2015).

Hitherto, LDET catalyzed by microorganisms has been observed for marine sediments (Malkin *et al.*, 2014), seasonal hypoxic basins (Seitaj *et al.*, 2015), salt marshes (Larsen *et al.*, 2014; Malkin *et al.*, 2014) and a freshwater stream (Risgaard-Petersen *et al.*, 2015), all representing saturated sediment environments rich in organic carbon. Inspired by recent reports on high abundances of not further classified *Desulfobulbaceae* at the fringes of a hydrocarbon contaminant plume (Winderl *et al.*, 2008; Pilloni *et al.*, 2011; Larentis *et al.*, 2013), we hypothesize that LDET might occur in freshwater aquifers fulfilling important ecological functions. Evidence supporting this hypothesis is provided here by laboratory incubations of aquifer sediments and by screening for cable bacteria across a redox gradient *in situ* in a tar-oil-contaminated aquifer.

## Materials and methods

### Site description and sampling

Sediment was sampled from a tar-oil-contaminated aquifer at a former coal gasification plant in Düsseldorf-Flingern, Germany (51°13'20.9"N 6°49'05.6"E). The site holds rather homogeneous quaternary sediment containing medium fine sand with grain sizes between 0.2 and 0.7 mm, a porosity of 0.34, and total organic carbon concentrations of 0.2–0.6% (dry weight; Anneser *et al.*, 2008; Anneser *et al.*, 2010). The aquifer is contaminated by a mixture of mono- and polycyclic aromatic hydrocarbons. Reduced inorganic sulfur varied in a previous study between 0.6 and 15.6 μmol g^−1^ (Anneser *et al.*, 2010). The groundwater table was located at ~6.5 m below surface in 2013; however, previous measurements between 2006 and 2009 indicated a dynamic groundwater table with fluctuations of up to 20 cm per year (Einsiedl *et al.*, 2015). A high-resolution multilevel monitoring well has been installed previously, allowing for groundwater sampling at 3 cm vertical resolution (Anneser *et al.*, 2008). Redox gradients and sediment microorganisms within the hydrocarbon plume were characterized in detail, previously (Schmitt *et al.*, 1996; Anneser *et al.*, 2008; Winderl *et al.*, 2008; Pilloni *et al.*, 2011).

For the present study, sediment samples were taken in September 2008 for incubation experiments in the laboratory and September 2013 for high-resolution community profiling. Core liners of 1 m each were retrieved over depths from 6 to 11 m below surface by hollow-stem auger drilling. The liners were opened by cutting with an angle grinder in the field under an argon atmosphere in order to minimize oxygen exposure. Sediment subsamples (~10 g each) were sampled at 3–10-cm resolution from the liners in 2013 and stored at −20 °C for later DNA extraction. In addition, sediments of different depths were transferred to sterile anoxic tap water, which was autoclaved under nitrogen atmosphere at 121 °C for 20 min. The sediments were stored at 12 °C under nitrogen for later batch experiments in the laboratory. For fluorescence *in situ* hybridization (FISH), sediments were sampled in 2013 from 10 cm above to 10 cm below the groundwater table. Aliquots of 0.5-g sediment were taken in 1-cm resolution along the core, directly transferred to either 4% paraformaldehyde or 2.5% glutardialdehyde and stored at 4 °C.

Groundwater was sampled in 3-cm resolution from the previously installed high-resolution multilevel well via steel capillaries connected to multichannel peristaltic pumps (Anneser *et al.*, 2008). The retrieved groundwater from each depth was subsampled in the field and treated for chemical analyses on site.

### Direct analyses of field samples

For dissolved sulfide analyses, 200 μl of sample were fixed in 1 ml of 2% (w/v) Zn-acetate solution. Sulfide concentrations were determined within 3 h after sampling, following the protocol of Cline (1969)). Fe(II) concentrations of groundwater samples were measured by the ferrozine assay (Stookey, 1970) as previously described (Anneser *et al.*, 2008). Toluene was measured by headspace analysis on a GC-MS (Thermo Electron, Dreieich, Germany) as previously described (Anneser *et al.*, 2008). Redox potentials were measured in the field directly after sampling with field redox electrodes (SenTix, WTW, Weilheim, Germany). Concentrations of nitrate and sulfate of field samples were measured with ion chromatography (Dionex DC-100, Idstein, Germany) as previously described (Anneser *et al.*, 2008). Intracellular ATP concentration of aquifer microbes was determined as proxy for viable microbial biomass in water and sediment samples immediately after sampling according to a modified protocol (Hammes *et al.*, 2010) of the BacTiter-Glo Microbial Cell Viability Assay (G8231; Promega, Mannheim, Germany). As correction for extracellular ATP in groundwater, samples were filtered (pore size 0.1 μm) and then measured as described above. Intracellular ATP was calculated according to: intracellular ATP=total ATP−extracellular ATP. For sediment samples only total ATP concentrations were measured and no correction for extracellular ATP was applied. Cell numbers were calculated from intracellular ATP concentrations according to Hammes *et al.* (2010), who reported an average amount of 1.75 × 10^−10^ nmol ATP per cell (Hammes *et al.*, 2010).

### Laboratory sediment incubations

Laboratory sediment incubations were prepared in 100-ml glass syringes (Fortuna Optima, Poulten und Graf, Wertheim, Germany). The syringes were filled with 40–50-ml sediment, which was homogenized by stirring with 2 μmol g^−1^ (wet weight) freshly precipitated, solid FeS. The addition of electron donor FeS was necessary because of de-watering of sediment cores during sampling, which also removed natural electron donors such as dissolved organic carbon or sulfide. To equilibrate, the columns were percolated twice with three pore volumes of anoxic, sterile (autoclaved under nitrogen for 20 min at 121 °C) tap water (80 ml), removing residual oxygen and dissolved ions from FeS synthesis. This water typically contains ~100 μM NO_3_^−^ and ~200 μM SO_4_^2−^. The water table was adjusted through the bottom outlet until it was ~1 mm above the sediment surface. Each of four replicate columns was incubated at 20 °C in the dark for up to 4 months. Duplicate abiotic control columns were amended with FeS, autoclaved under nitrogen for 30 min at 121 °C and treated similar to the biotic incubations afterward. FeS was synthesized freshly in the laboratory by mixing equal volumes of 1 M FeCl_2_ and 1 M Na_2_S dissolved in anoxic MilliQ water. The precipitated black FeS was washed twice with anoxic MilliQ water in order to remove any sodium and chloride ions released during synthesis.

### Column biogeochemistry

Microprofile oxygen in columns was measured by microsensors with a tip size of 0.1 mm (Revsbech, 1989; Unisense, Aarhus, Denmark) in 0.25-mm resolution. Porewater pH was measured in 1-mm resolution with a Perphect Ross Micro Combination pH electrode (Thermo Fisher Scientific Inc., Waltham, MA, USA) with a tip size of 3 mm connected to a pH meter (WTW series pH 730, InoLab, Weilheim, Germany) with a motorized micromanipulator (Unisense) for depth adjustment. The pore water chemistry in replicate columns developed in a similar way and fluxes associated with LDET were reproducible (Table 1 and Supplementary Figure S1). After 2.5 and 3 months of column incubation, two replicate sediment cores were shock-frozen in liquid nitrogen and cut into 3–12 mm slices with a circular diamond blade (Proxxon, Föhren, Germany). After thawing, pore water for sulfide measurements was collected with an automatic pipette in an oxygen-free glove box. Sulfide was measured with a colorimetric assay (Cline, 1969), which was downscaled to a reaction volume of 2 ml and a corresponding sample size of 40 μl. Three aliquots of each reaction were analyzed at 660 nm on a Wallac 1420 Viktor3 plate reader (Perkin Elmer, Waltham, MA, USA). Sulfide concentrations were derived by linear regression from a Na_2_S standard curve with a calibration range between 10 μM and 10 mM. Ion chromatography was not conducted for laboratory incubations because of limited amounts of recovered pore water. Porewater alkalinity was determined by titration with 0.1 M HCl (APHA *et al.*, 1998) of the anoxic tap water that was used for equilibration.

Cathodic proton consumption, diffusive oxygen uptake and cathodic oxygen consumption were calculated from geochemical gradients as previously described (Nielsen *et al.*, 2010; Risgaard-Petersen *et al.*, 2012). Diffusive fluxes were calculated according to the formula:


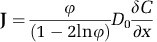


where *ϕ* is the sediment porosity of 0.34 (Anneser *et al.*, 2010); *D*_0_ is the molecular diffusion coefficient derived from literature (Cussler, 2009) and adjusted to a temperature of 20 °C assuming a linear correlation with temperature according to the Stokes–Einstein equation. This led to diffusion coefficients at 20 °C for O_2_, HCO_3_^−^, CO3^2−^, OH^−^ and H^+^ of 2.06, 1.16, 0.94, 5.51 and 9.14 (each multiplied by 10^−5^ cm^2^ s^−1^), respectively. *C* is the concentration and *x* the depth. Cathodic proton consumption was calculated as the sum of alkalinity fluxes above and below the pH maximum. Alkalinity fluxes were calculated from diffusive fluxes of individual compounds contributing to alkalinity as previously described (Malkin *et al.*, 2014) by assuming constant values of dissolved inorganic carbon with depth according to the formula:


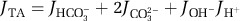


Current densities were determined from cathodic proton consumption (CPC) by using a conversion factor of 1.036 × 10^−5^ mol e^−1^ s^−1^ A^−1^. Assuming a consumption of four protons per oxygen molecule reduced by LDET, cathodic oxygen consumption was derived from: COC=CPC × 0.25, where COC is cathodic oxygen consumption.

### DNA extraction and molecular methods

DNA from sediment of batch and field samples was extracted in triplicates each with 1 g sediment according to a previously described protocol (Winderl *et al.*, 2008). For terminal-restriction fragment length polymorphism (T-RFLP) analysis, the 16S rRNA gene was amplified with primers Ba27f–FAM and 907r as described (Pilloni *et al.*, 2011). The PCR conditions were as follows: initial denaturation at 94 °C (5 min), followed by 28 cycles of denaturation (94 °C; 30 s), annealing (52 °C; 30 s) and elongation (70 °C; 60 s) and a final elongation (70 °C; 5 min). Fam-labeled amplicons (80 ng) were digested with 0.3 μl MSPI in Tango buffer with 10 μl total reaction volume at 37 °C for 2 h. Fragments were desalted with the DyeEx 2.0 Spin Kit (Qiagen, Hilden, Germany). One microliter of the desalted fragments was mixed with 13 μl HiDi Formamide (Thermo Fisher Scientific Inc.), which contained a 1/300 dilution of MapMarker-100 ROX Size Standard. Fragments was denatured for 5 min at 95 °C and analyzed using capillary electrophoresis on ABI 3730 DNA analyzer (Thermo Fisher Scientific Inc.). Data were evaluated with the Gene Mapper software 5.1 (Thermo Fisher Scientific Inc.) and further processed with the T-REX online tool for T-RFLP analysis (Culman *et al.*, 2009). Peak heights were normalized and the T-RF clustering threshold was adjusted to 2 bp. The mean relative T-RF abundances and standard deviations were calculated from fingerprints of triplicate DNA extractions.

Amplicon pyrosequencing, data processing and taxon assessment were performed as previously published (Pilloni *et al.*, 2011). Maximum likelihood phylogenetic trees of 16S rRNA genes were constructed with the MEGA6 (Tamura *et al.*, 2013) software (Version 6.06) by creating an alignment of representative sequences with the MUSCLE algorithm (Edgar, 2004). The chosen amplicon contigs were deposited at GenBank under the accession numbers KR262833 to KR262839. All sequencing reads are deposited at SRA under study accession number SRP063036.

### FISH analysis

On the basis of 16S rRNA sequences derived from assembled sequence read contigs, as well as on previously published full-length 16S rRNA sequences (Kunapuli *et al.*, 2008), a specific FISH probe FliDSB194 (Supplementary Table S1) was designed for *Desulfobulbaceae* from the Flingern site using the ARB software package (Ludwig *et al.*, 2004; Supplementary Table S2). Fresh sediment samples from laboratory and field were mixed 1:1 with either 4% paraformaldehyde or 2.5% glutardialdehyde and stored at 4 °C until further processing. The sediment was cautiously washed 1:1 in phosphate-buffered saline buffer to release filaments to the supernatant. Twenty microliters of supernatant were then diluted in 200 μl MilliQ water, mixed by flipping and pipetted in 40-μl aliquots to eight-well microscope slides. The slides were dried for 90 min at 46 °C. Afterward, they were dehydrated by successively increasing ethanol concentrations of 50, 80 and 99% for 3 min each. Hybridization was performed for 2.5 h, followed by a 30-min washing step according to a previously published protocol (Pernthaler *et al.*, 2001). Specific oligonucleotide probes DSB706 (Loy *et al.*, 2002) and FliDSB194 were used for the detection of filamentous *Desulfobulbaceae* at a hybridization stringency of 35% formamide (Supplementary Table S2). Cells were counterstained with 4',6-diamidino-2-phenylindole and embedded in Citiflour. Labeled cells were detected with fluorescence microscopy (Axioskop 2 plus, Zeiss, Jena, Germany) by using specific filters for 6-Fam (DSB706), Cy3 (FliDSB194) and 4',6-diamidino-2-phenylindole (counterstained cells). For imaging, pictures were taken with the digital camera AxioCam HRm (Zeiss) and the software AxioVision (Version 4.8.2; Zeiss).

## Results

### LDET and cable bacteria in laboratory sediment columns

**Figure 1:**
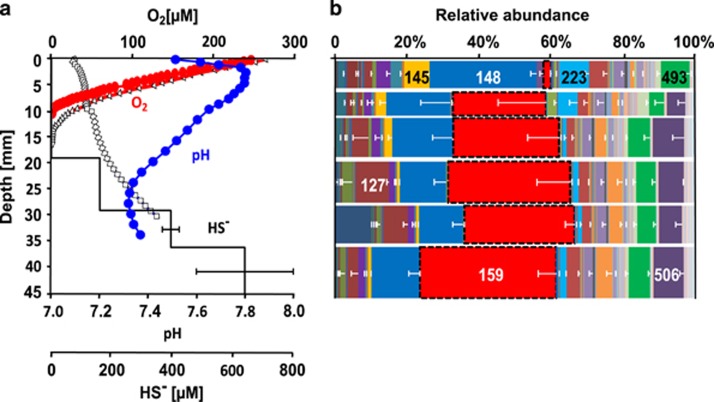
Geochemical gradients and microbial community analysis from a representative incubation of sediments taken from the Flingern aquifer. (**a**) Porewater profiles of O_2_, pH and HS^−^ of homogenized sediment amended with 2 μmol g^−1^ sediment after 70 days of incubation in the dark. One typical replicate out of four is shown. Error bars for sulfide concentrations in different slices of the cut sediment represent s.d.'s of three technical replicates. Open symbols show pore water profiles of O_2_ (triangles) and pH (squares) of one typical abiotic control column out of two. (**b**) Corresponding depth-resolved relative abundance of T-RF 159 (dashed frame) representing members of the *Desulfobulbaceae* as the mean values of three independent DNA extractions. Error bars show s.d.'s of relative abundances determined from triplicate DNA extractions of one typical replicate out of two.

To demonstrate the potential occurrence of LDET in aquifers, sediments from a previously investigated tar oil-contaminated aquifer in Düsseldorf, Germany, were amended with 2 μmol g^−1^ FeS, saturated with anoxic water and incubated at 20 °C in the dark in open-top glass syringes. After 70 days, microsensor measurements in pore water showed that oxygen was penetrating 8±2 mm (O_2_<0.3 μM) into the sediment (Figure 1a and Table 1). The pH increased to 7.8 within the oxic zone, in accordance with proton consumption by cathodic oxygen reduction. Beneath the oxygen minimum, a suboxic zone of at least 9 mm separated the oxic and sulfidic zones. The coarse resolution of the sulfide profile did not allow for an exact determination of the suboxic–sulfidic transition and might underestimate the width of the suboxic zone. Here, the pH continuously decreased toward a minimum of pH 7.3 within the sulfidic zone at 27.5 mm, in accordance with proton release by anodic sulfide oxidation. The geochemical profiles thus indicated that sulfide oxidation is connected to oxygen reduction over a distance of 15–25 mm (Figure 1a). Calculation of oxygen and alkalinity fluxes from gradient slopes (Nielsen *et al.*, 2010; Malkin *et al.*, 2014) indicated a cathodic oxygen consumption of 340±67 μmol m^−2^ per day and a current density of 1.5±0.3 mA m^−2^ (Table 1). This corresponds to a theoretical sulfate recycling of 170±34 μmol m^−2^ per day within the sulfidic zone, assuming complete oxidation of sulfide to sulfate and evenly distributed dissolved inorganic carbon of 5.5 mM as pH buffer over the entire column. In contrast, autoclaved control columns did not show an increase but a 0.7 lower pH in the oxic zone, which was most likely caused by chemical FeS oxidation (Figure 1a). Solution of CO_2_ from the atmosphere might also have led to acidification as the water was degassed during the sterilization by autoclaving. In addition, abiotic oxygen profiles differed significantly from active incubations by deeper oxygen penetration of 18±2 mm and a corresponding oxygen flux of 513±85 μmol m^−2^ per day after 70 days of incubation compared with 950±360 μmol m^−2^ per day for active incubations (Figure 1a and Table 1).

**Figure 2:**
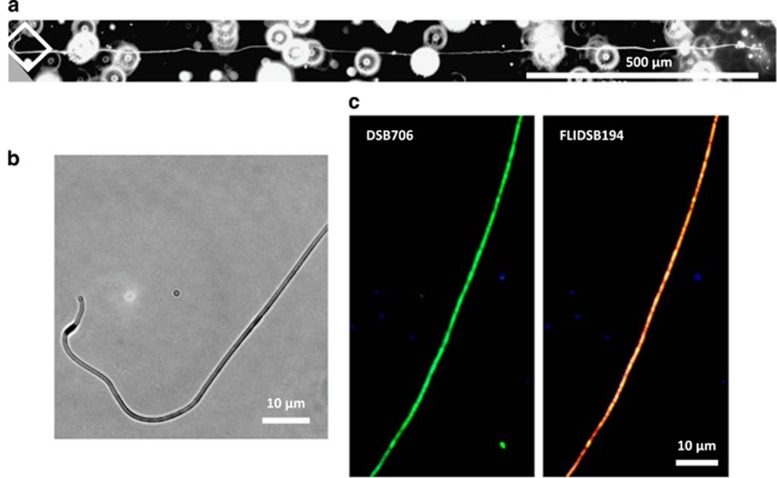
(**a**) Microscopic image of a filament fragment of ~1.7 mm length. Three pictures were merged to cover the full length of the fragment. The white square indicates the part of the filament shown in higher magnification in (**b**). (**c**) Micrographs of filaments stained with FISH probes specific for the family *Desulfobulbaceae* (DSB706; 6-FAM labeled) and for groundwater cable bacteria (FLIDSB194, Cy3 labeled). Each image is presented as an overlay of two pictures taken with filters for specific probe fluorescence and 4',6-diamidino-2-phenylindole for counterstaining.

After completing the geochemical measurements, columns were sacrificed and sediment cores cut into 3–12 mm slices. Long filamentous bacteria were discovered in the suboxic zone of these sediments using light microscopy. Filament fragments were up to 5.5 mm long and between 0.4 and 1.5 μm in diameter (Figure 2).

In order to elucidate the distribution of these bacteria in the sediment columns and their phylogenetic affiliation, T-RFLP and amplicon pyrosequencing were conducted. T-RFLP fingerprinting showed a T-RF of 159 bp length as predominant throughout the columns, with a relative abundance of up to 40% throughout the suboxic and sulfidic zones, where LDET occurred (Figure 1b). Sequencing of 16S rRNA gene fragments from extracted DNA showed *Desulfobulbaceae* to represent up to 34% of all sequencing reads within the anoxic part of the sediment. The comparable abundance of T-RF 159 and *Desulfobulbaceae* sequences —together with a predicted cut size of 162 bp— identified T-RF 159 as member of the family *Desulfobulbaceae* (Supplementary Figure S2). These molecular techniques included amplification steps of 16S genes and therefore can only provide relative abundances within the bacterial community and do not reflect absolute cell abundances. Sequence similarity to 16S rRNA genes of marine (Schauer *et al.*, 2014) and freshwater (Risgaard-Petersen *et al.*, 2015) cable bacteria was only up to 88%. The closest cultivated relative with 91% similarity was *Desulfurivibrio alkaliphilus* AHT2, a facultative chemolithoautotrophic, non-sulfate-reducing bacterium (Sorokin *et al.*, 2008).

To verify the placement of the filaments within the *Desulfobulbaceae* sequences mentioned above, a specific FISH probe (FliDSB194) was designed and applied to sediment from laboratory incubations (Figure 2c). The long filamentous bacteria, previously observed with phase contrast microscopy, specifically hybridized with this probe, as well as with the *Desulfobulbaceae*-specific probe DSB706 (Loy et al., 2002; Figure 2c).

### Possible LDET at the capillary fringe *in situ*

**Figure 3:**
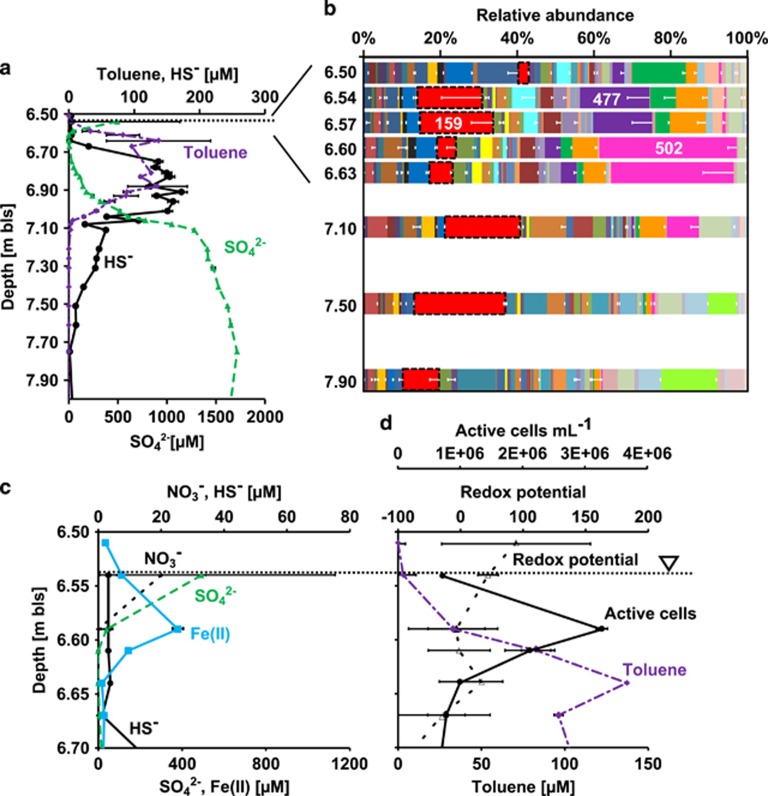
Depth profiles of geochemistry of water samples from a high-resolution monitoring well installed in Flingern and of microbial community compositions from a sediment core drilled at 1 m distance. (**a**) Groundwater chemistry of duplicate measurements showing the zone of toluene contamination as well as the sulfide- and sulfate concentrations. (**b**) T-RFLP microbial community fingerprints obtained from triplicate DNA extractions of different sediment layers. T-RF 159 (dashed frame) is representing members of the *Desulfobulbaceae*. Data show the mean values and s.d.'s of three independent DNA extractions. (**c**, **d**) Geochemistry and active cell densities estimated from intracellular ATP concentrations of groundwater at the upper plume fringe. Bls, below land surface. The dashed horizontal lines indicate the groundwater table.

Sampling of groundwater at 3-cm-depth resolution was performed at the same tar oil-contaminated aquifer in Düsseldorf, Germany, to search for indications of LDET *in situ*. The toluene plume ranged from the groundwater table at 6.54 m below surface (bls) down to 7.21 m (Figure 3a). Nitrate and sulfate were detectable at the upper plume fringe at 6.54 bls, but nitrate decreased below the detection limit at 6.59 m and sulfate at 6.62 m bls. (Figure 3c). The steep geochemical gradients and the depletion of electron acceptors in the plume core suggested strong microbial toluene degradation at the plume fringes (Anneser *et al.*, 2008; Winderl *et al.*, 2008; Larentis *et al.*, 2013). A steep decrease in sulfate from the lower plume fringe toward the plume core indicated sulfate reduction as a predominant process between 6.90 and 7.10 m bls. Highest ferrous iron concentrations of 380 μM and the highest number (3 × 10^6^ cells/ml) of active bacteria estimated from intracellular ATP concentrations were also detected at the upper plume fringe at 6.59 m bls (Figure 3d). Such ferrous iron concentrations could be because of microbial biodegradation coupled to iron reduction or an oxygen- or nitrate-dependent release of ferrous iron from iron sulfides by LDET within the suboxic zone.

**Figure 4:**
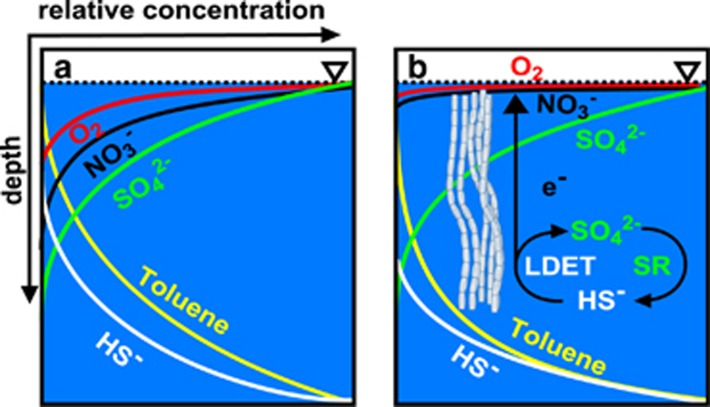
Conceptual model of the impact of LDET by cable bacteria on sulfur cycling and contaminant degradation at the plume fringe. (**a**) Redox zonation at a toluene plume fringe only controlled by dispersive/diffusive mixing in the absence of cable bacteria. (**b**) A plume fringe scenario including sulfide re-oxidation by cable bacteria leads to higher availability of sulfate for toluene degradation by sulfate reduction (SR) and a broader zone of biodegradation at the plume fringes. Steeper gradients indicate higher fluxes of solutes.

At 1 m distance from the high-resolution monitoring well, we took intact sediment cores and studied the microbial communities in 3 cm vertical intervals. Microbial community fingerprinting of DNA extracted from sediment samples showed relatively high abundances of 16% of the same 159 bp T-RF up to the capillary fringe at 6.54 m bls and 22% at the lower plume fringe at 7.10 and 7.50 m bls (Figure 3b). Sequencing of 16S rRNA gene fragments over depth substantiated this T-RF to represent the filamentous *Desulfobulbaceae* detected in the laboratory incubations (Figure 4). Other abundant sequences found at the capillary fringe and upper plume fringe belonged to *Ignavibacteria* (T-RF 163) and *Thiobacillus denitrificans* (T-RF 477). Dominant taxa within the plume core were *Rhodocyclaceae* (T-RF 502) and *Desulfobulbaceae* related to the toluene-degrading strain TRM1 (Meckenstock, 1999) at the lower fringe, previously identified as the key toluene degraders in this aquifer (Pilloni *et al.*, 2011; Supplementary Figure S2).

## Discussion

Recently, filamentous cable bacteria were discovered in organic-rich marine sediments to spatially bridge the redox half reactions of sulfide oxidation and oxygen reduction via LDET over 1–2 cm (Nielsen *et al.*, 2010; Pfeffer *et al.*, 2012; Malkin *et al.*, 2014). Here, we aimed to investigate whether microbially mediated LDET may also occur in freshwater sediments, specifically in hydrocarbon-contaminated groundwater, where it could recycle sulfate as electron acceptor and thus increase biodegradation rates.

In FeS-amended laboratory incubations of sediments from the investigated site, a suboxic zone developed with no detectable oxygen or sulfide but with distinct cathodic pH maxima and anodic pH minima indicative of LDET (Nielsen *et al.*, 2010). Oxygen only penetrated 8 mm into the sediment and yet served as a direct sink for electrons from oxidation of sulfide up to 19 mm below, congruent with the LDET hypothesis. The calculated current density between the oxic and the anoxic layers was higher than 1.5 mA m^−2^ corresponding to a cathodic oxygen consumption of 340 μmol m^−2^ per day and representing 40% of the total oxygen consumption. This calculation does not include calcite precipitation and ferrous iron oxidation and might therefore underestimate cathodic oxygen consumption (Risgaard-Petersen *et al.*, 2014). Moreover, the coarse sediment did not allow for microsensor measurements of pH profiles, but only for a macroelectrode with a tip size of 3 mm at a resolution of 1 mm. This smoothened the pH profile, resulting in lower calculated alkalinity fluxes and cathodic oxygen consumption. Thus, the electron transfer rate inferred for groundwater cable bacteria was 1–2 orders of magnitude lower than the 4.6–92 mA m^−2^ reported for marine sediments (Nielsen *et al.*, 2010; Nielsen and Risgaard-Petersen, 2015). This discrepancy could be also caused—besides the underestimation of fluxes—by up to 100-fold lower sulfide concentrations in the investigated sediment compared with marine sediments (Rao *et al.*, 2015).

16S rRNA gene sequencing and FISH identified groundwater cable bacteria as members of the *Desulfobulbaceae*. The marine cable bacteria are only distantly related with 88% similarity on the 16S rRNA level. The closest cultivated relative with 91% 16S rRNA gene similarity was *Desulfurivibrio alkaliphilus* AHT 2, which can grow chemo–litho–autotrophically with H_2_ as electron donor. The strain cannot perform sulfate reduction but utilizes elemental sulfur, thiosulfate, and nitrate as electron acceptor (Sorokin *et al.*, 2008). The genome exhibits a complete aerobic respiratory chain with a terminal cytochrome *c* oxidase cbb3 type. Sox genes for sulfide oxidation are not present, but a reverse sulfate reduction pathway seems possible. The second-most closely related chemo–litho–autotrophic organism, the arsenate-reducing strain MLMS-1, is able to grow with sulfide as electron donor and shows 90% 16S rRNA gene similarity to the groundwater cable bacteria (Hoeft *et al.*, 2004).

The high abundance of cable bacteria at the oxic–anoxic interface of aquifers indicates an ecological competitiveness to other sulfide-oxidizing, chemo–litho–autotrophs such as, for example, *Thiobacillus*. A possible explanation is fluctuations of the groundwater table leading to shifting redox gradients at the capillary fringe (Meckenstock *et al.*, 2015). Under such dynamic conditions, unicellular microorganisms will often not be located in the zone of overlapping countergradients and lack either electron donor or acceptor. Such unfavorable conditions can be overcome by spatially decoupling of the oxidation and reduction half reactions by cable bacteria. Only some cells at both ends of the filaments need to have access to electron donors or acceptors, respectively. Thus, cable bacteria could still be active as long as the spatial shifts of redox gradients do not exceed the lengths of the filaments. Even though groundwater table fluctuations of ~20 cm within 1 year have been reported from this site (Einsiedl *et al.*, 2015), cable bacteria could still have a competitive advantage by buffering short-term fluctuations in a smaller scale. Cable bacteria can also adapt rapidly to changing conditions, as shown for laboratory incubations (Nielsen *et al.*, 2010) and seasonal hypoxic basins (Seitaj *et al.*, 2015). The longest filament that we could find microscopically was ~5 mm. However, natural filaments and cable networks that are not disrupted by sampling could be much longer. For marine sediments, fragment lengths of filaments up to 1.5 cm have been reported (Pfeffer *et al.*, 2012).

The detection of LDET *in situ* remains a difficult task. Our monitoring well provided water samples at 3 cm vertical resolution. Although this is probably the highest resolution for water sampling in aquifers of that depths reported to date, it is obviously not sufficient to record pH profiles at the mm range, which might be necessary to map LDET *in situ*. Moreover, pH profiles could also not be determined from fresh sediment cores, as cores commonly de-water upon retrieval during drilling, prohibiting the reconstruction of pore water geochemistry. Thus, analyzing sediment cores with molecular and microscopic tools is the only reliable way for detecting cable bacteria in aquifers so far. However, LDET might potentially be traced *in situ* by remotely assessing associated electric fields (Revil *et al.*, 2010; Risgaard-Petersen *et al.*, 2014; Revil *et al.*, 2015). In fact, our results provide the first field evidence for biogeobatteries in aquifers comprising cable bacteria as electron conductors (Revil *et al.*, 2010). By oxidizing sulfide, groundwater cable bacteria resemble the anode of a microbial fuel cell. Revil *et al.* (2015) showed direct oxidation of propylene glycol by electric currents through a conductive iron body in laboratory experiments (Revil *et al.*, 2015). Such electric currents create electric potential anomalies, which might be a good monitoring tool for localizing hotspots of LDET *in situ* (Naudet *et al.*, 2004; Atekwana and Slater, 2009; Revil *et al.*, 2010; Revil *et al.*, 2015). However, the direct link between LDET and the observation of electric potentials at contaminated sites still remains to be proven.

Our field geochemical data indicated that oxygen and nitrate were at least 6 cm apart from detectable dissolved sulfide. As only distances below 3 cm for LDET have been reported so far, LDET might be fueled by other reduced sulfur compounds as electron donor such as precipitated FeS. This would be supported by our laboratory incubations where FeS turned out to be an excellent electron donor for LDET. It is also likely that sulfide produced by sulfate-reducing toluene degradation is immediately re-oxidized by LDET (Figure 4b) or precipitated as FeS in the field. In fact, a previous study conducted at the same site demonstrated strong sulfur cycling at the upper plume fringe, the place of LDET reported here (Einsiedl *et al.*, 2015).

The discovery of LDET catalyzed by cable bacteria in laboratory incubations with sediments from hydrocarbon-contaminated aquifers might provide a new perspective of microbial activities at the capillary or plume fringes. Even though the presence of cable bacteria at the plume fringes provides a first evidence for LDET *in situ,* the quantitative impact on the biogeochemistry and contaminant degradation remains to be investigated. The electric shortcut by the filaments could strongly increase electron fluxes (Risgaard-Petersen *et al.*, 2014) across redox interphases (Figure 4). Thus, it could extend the recently established plume fringe concept (Cirpka *et al.*, 1999; Mayer *et al.*, 2001; Thornton *et al.*, 2001; Maier and Grathwohl, 2006; Anneser *et al.*, 2008; Bauer *et al.*, 2008; Winderl *et al.*, 2008; Meckenstock *et al.*, 2015), which states that electron acceptors are depleted in the core of contaminant plumes. Biodegradation is accordingly restricted to the plume fringes where electron acceptors are supplied from the outside by dispersion or diffusion (Figure 4a). Recycling of sulfate by LDET at the plume fringes might overcome the spatial separation of electron donors and acceptors to a certain extent and consequently lead to an enhancement of biodegradation as compared with a system otherwise fully controlled by dispersion (Figure 4b; Anneser *et al.*, 2008; Bauer *et al.*, 2008; Anneser *et al.*, 2010; Pilloni *et al.*, 2011).

## Figures and Tables

**Table 1:** Geochemical parameters associated with LDET detected in batch incubations

	*OPD (mm)*	*DOU (μmol m*^−*2*^ *per day)*	*COC (μmol m*^−*2*^ *per* *day*)	*Current density (mA m*^−*2*^*)*
Active incubation (*n*=4)	8±2	950±360	340±67	1.5±0.3
Abiotic control (*n*=2)	18±2	513±95	NA	NA

Abbreviations: COC, cathodic oxygen consumption; DOU, diffusive oxygen uptake; LDET, long-distance electron transfer; NA, not applicable; OPD, oxygen penetration depth.

Signal of LDET in four replicate cores in comparison with two abiotic control columns after 70 days of incubation.
