# Supplementary material for: Long-distance electron transfer by cable bacteria in aquifer sediments
Source: ISME J. 2016 Apr 8;10(8):2010–9. doi: 10.1038/ismej.2015.250 (PMC4939269; doi:10.1038/ismej.2015.250)
Supplement: Supplementary Table S1 [file ismej2015250x1.doc]

**Table S1**: Oligonucleotide probes used for FISH

| **Probe** | **Sequence** | **Label** | **Target organisms** |
| --- | --- | --- | --- |
| **DSB706** | 5’-ACCGGTATTCCTCCCGAT-3’ | 6-Fam | Most *Desulfobulbaceae* |
| **FliDSB194** | 5’-GGAGAGGTCTCCTTTCCTTA-3’ | Cy3 | Groundwater cable bacteria |
