# Supplementary material for: Long-distance electron transfer by cable bacteria in aquifer sediments
Source: ISME J. 2016 Apr 8;10(8):2010–9. doi: 10.1038/ismej.2015.250 (PMC4939269; doi:10.1038/ismej.2015.250)
Supplement: Supplementary Table S2 [file ismej2015250x2.doc]

**Table S2**: Specificity of probe FliDSB194

| **Sequence** | **Accession No.** | **MM** | **Alignment** |
| --- | --- | --- | --- |
| Probe FliDSB194 |  |  | 3’-ATTCCTTTCCTCTGGAGAGG-5’ |
| Target |  |  | 5’-TAAGGAAAGGAGACCTCTCC-3’ |
| BF enrichment *Desulfobulbaceae* clone HT06Ba23 | EU016449 | 0 | ∙ ∙ ∙ ∙ ∙ ∙ ∙ ∙ ∙ ∙ ∙ ∙ ∙ ∙ ∙ ∙ ∙ ∙ ∙ ∙ |
| Flingern enrichment culture clone 13Fcon08 | HQ625672 | 0 | ∙ ∙ ∙ ∙ ∙ ∙ ∙ ∙ ∙ ∙ ∙ ∙ ∙ ∙ ∙ ∙ ∙ ∙ ∙ ∙ |
| Lake water clone: rS35m_60 | AB754079 | 0 | ∙ ∙ ∙ ∙ ∙ ∙ ∙ ∙ ∙ ∙ ∙ ∙ ∙ ∙ ∙ ∙ ∙ ∙ ∙ ∙ |
| Trichloroethene-contaminated site clone FTLpost26 | AF529133 | 0 | ∙ ∙ ∙ ∙ ∙ ∙ ∙ ∙ ∙ ∙ ∙ ∙ ∙ ∙ ∙ ∙ ∙ ∙ ∙ ∙ |
| *Desulfobacterales* bacterium clone SFeT25 | JQ723604 | 0 | ∙ ∙ ∙ ∙ ∙ ∙ ∙ ∙ ∙ ∙ ∙ ∙ ∙ ∙ ∙ ∙ ∙ ∙ ∙ ∙ |
| Aquifer sediment clone EMIRGE_OTU_s3t2d_674 | JX222667 | 0 | ∙ ∙ ∙ ∙ ∙ ∙ ∙ ∙ ∙ ∙ ∙ ∙ ∙ ∙ ∙ ∙ ∙ ∙ ∙ ∙ |
| Kristineberg tailing dump clone 6_92_63|1|Lib6 | KJ650741 | 0 | ∙ ∙ ∙ ∙ ∙ ∙ ∙ ∙ ∙ ∙ ∙ ∙ ∙ ∙ ∙ ∙ ∙ ∙ ∙ ∙ |
| *Desulfobacterales* bacterium clone SFeB33 | JQ723620 | 1 | ∙ G ∙ ∙ ∙ ∙ ∙ ∙ ∙ ∙ ∙ ∙ ∙ ∙ ∙ ∙ ∙ ∙ ∙ ∙ |
| Lake water clone: rS35m_72 | AB754082 | 2 | ∙ G ∙ ∙ ∙ ∙ ∙ ∙ ∙ ∙ T ∙ ∙ ∙ ∙ ∙ ∙ ∙ ∙ ∙ |
| Uranium mining mill tailing clone GR-Sh2-18 | AJ296571 | 2 | ∙ ∙ ∙ ∙ ∙ ∙ ∙ ∙ ∙ ∙ T ∙ ∙ ∙ ∙ ∙ ∙ ∙ T ∙ |
| *Desulfurivibrio alkaliphilus* strain AHT2 | EF422413 | 2 | ∙ ∙ ∙ ∙ ∙ ∙ ∙ ∙ ∙ ∙ T ∙ ∙ ∙ ∙ ∙ ∙ ∙ T ∙ |
| *Desulfobulbus sp.* DSM 2033 | EF442993 | 4 | ∙ ∙ ∙ ∙ ∙ ∙ ∙ ∙ ∙ ∙ T ∙ G ∙ ∙ ∙ ∙ ∙ G A |
| Marine cable bacteria | JX091072 | 6 | C ∙ ∙ ∙ _ ∙ ∙ ∙ ∙ ∙ T ∙ G ∙ ∙ ∙ ∙ ∙ A T |
|  |  |  |  |

Specificity of probe FliDSB194 for selected environmental and cultured sequences in Genbank. MM: Mismatches. Pairings are indicated as dots.
