# Supplementary material for: Long-distance electron transfer by cable bacteria in aquifer sediments
Source: ISME J. 2016 Apr 8;10(8):2010–9. doi: 10.1038/ismej.2015.250 (PMC4939269; doi:10.1038/ismej.2015.250)
Supplement: Supplementary Information [file ismej2015250x3.doc]

**Figure S1. A-D)** Porewater profiles of O_2_ and pH of homogenized sediment amended with 2 µmol g^-1^ FeS after 70 days of incubation in the dark. **E-F)** Porewater profiles of O_2_ and pH of abiotic control columns.

**Figure S2.** Maximum likelihood phylogenetic tree based on 16S rRNA sequences showing the phylogenetic affiliation of groundwater cable bacteria (red circles), marine cable bacteria (green squares), freshwater cable bacteria (violet triangles) and sequences related to the toluene-degrading strain TRM1 (blue diamonds) (Meckenstock, 1999). The prefix of sequence names indicate sequences derived from laboratory (lab-) batch incubations or directly from the field (Flingern-). The suffix indicates the sampling depth below sediment surface.
