# Supplementary figures and images for: Long-distance electron transfer by cable bacteria in aquifer sediments
Source: ISME J. 2016 Apr 8;10(8):2010–9. doi: 10.1038/ismej.2015.250 (PMC4939269; doi:10.1038/ismej.2015.250)

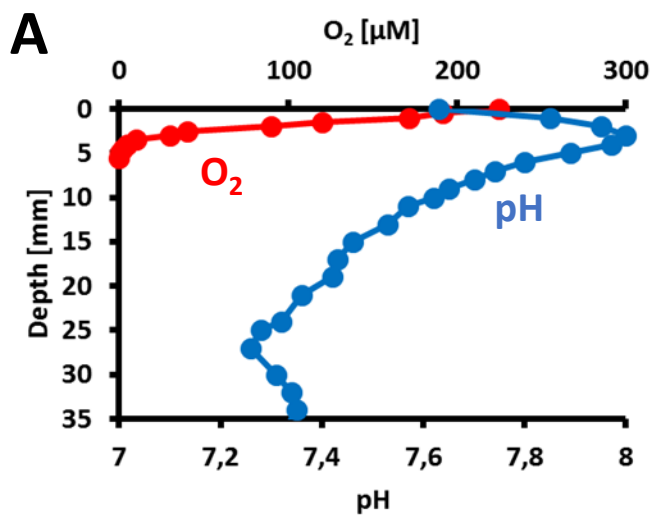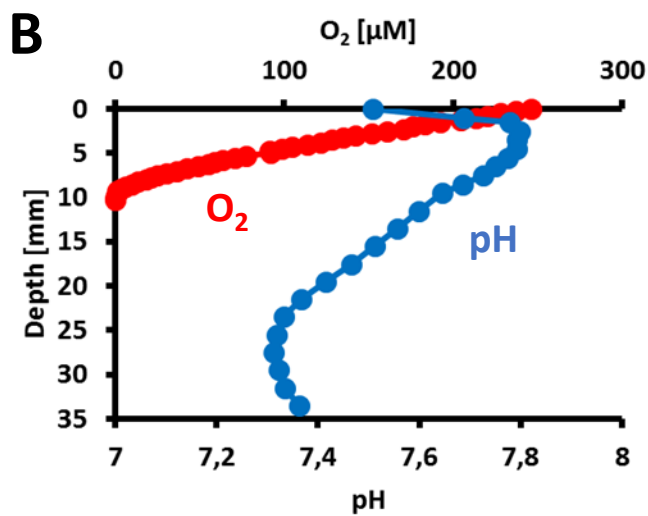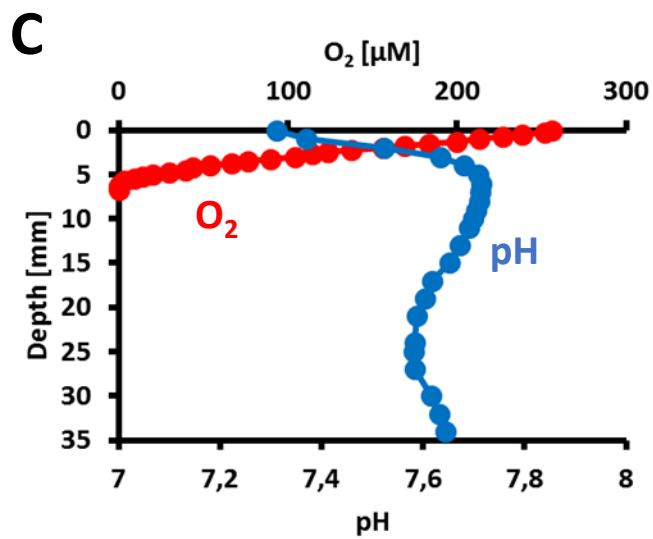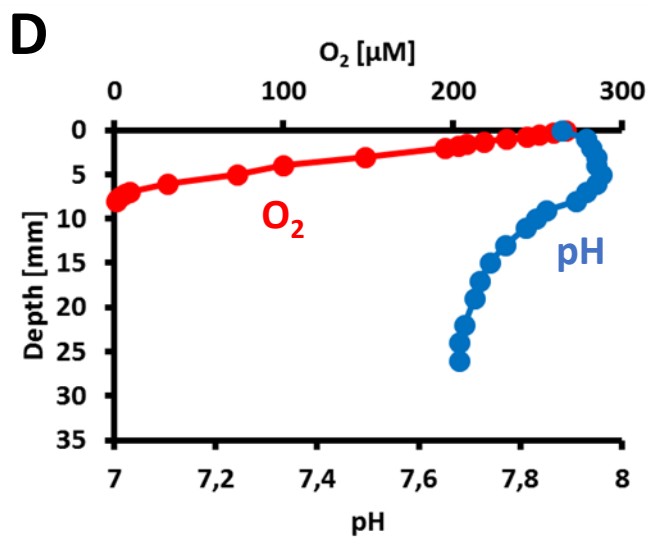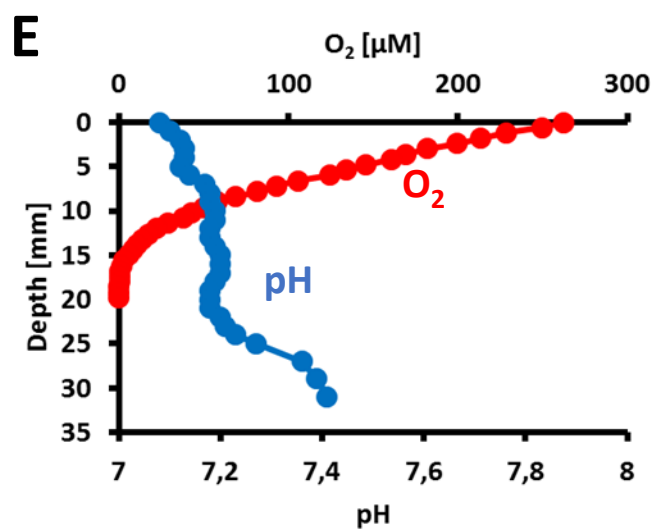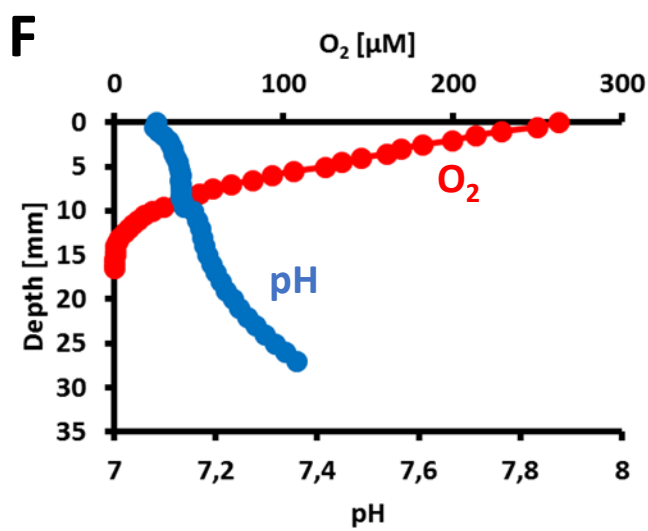

Supplement: Supplementary Figure S1 [file ismej2015250x4.pdf]

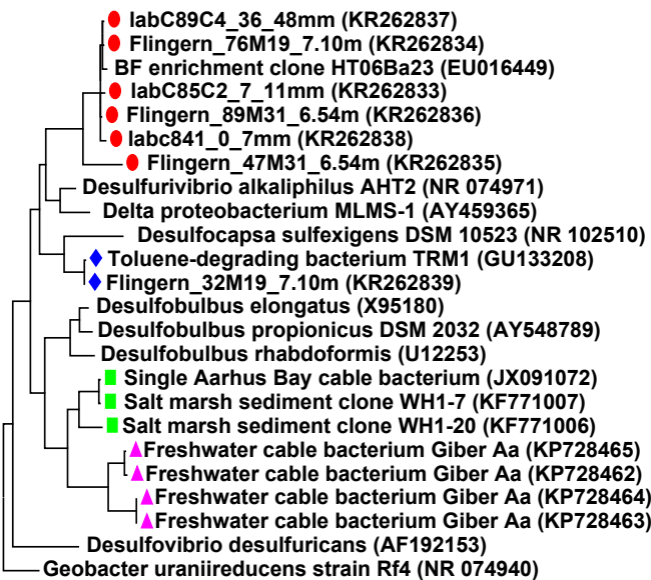

0.1

Supplement: Supplementary Figure S2 [file ismej2015250x5.pdf]
